# Identification of an Individualized Prognostic Signature Based on the RWSR Model in Early-Stage Bladder Carcinoma

**DOI:** 10.1155/2020/9186546

**Published:** 2020-06-04

**Authors:** Liyang Liu, Xiaodan Zhong, Haining Cui, Hao Zhang, Linyu Wang, Yuanning Liu

**Affiliations:** ^1^College of Physics, Jilin University, Changchun, Jilin, China; ^2^College of Computer Science and Technology, Jilin University, Changchun, Jilin, China; ^3^Department of Pediatric Oncology, The First Hospital of Jilin University, Changchun, Jilin, China

## Abstract

Bladder cancer (BLCA) is the fourth common cancer among males in the United States, which is also the fourth leading cause of cancer-related death in old males. BLCA has a high recurrence rate, with over 50% of patients which has at least one recurrence within five years. Due to the complexity of the molecular mechanisms and heterogeneous cancer feature, BLCA clinicians find it hard to make an efficient management decision as they lack reliable assessment of mortality risk. Meanwhile, there is currently no screening suitable prognostic signature or method recommended for early detection, which is significantly important to early-stage detection and prognosis. In this study, a novel model, named the risk-weighted sparse regression (RWSR) model, is constructed to identify a robust signature for patients of early-stage BLCA. The 17-gene signature is generated and then validated as an independent prognostic factor in BLCA cohorts from GSE13507 and TCGA_BLCA datasets. Meanwhile, a risk score model is developed and validated among the 17-gene signature. The risk score is also considered an independent factor for prognosis prediction, which is confirmed through prognosis analysis. The Kaplan-Meier with the log-rank test is used to assess survival difference. Furthermore, the predictive capacity of the signature is proved through stratification analysis. Finally, an effective patient classification is completed by a combination of the 17-gene signature and stage information, which is for better survival prediction and treatment decisions. Besides, 11 genes in the signature, such as coiled-coil domain containing 73 (CCDC73) and protein kinase, DNA-activated, and catalytic subunit (PRKDC), are proved to be prognosis marker genes or strongly associated with prognosis and progress of other types of cancer in published literature already. As a result, this paper would more accurately predict a patient's prognosis and improve surveillance in the clinical setting, which may provide a quantitative and reliable decision-making basis for the treatment plan.

## 1. Introduction

Bladder cancer (BLCA) is the fourth most common cancer for men in the United States, with an estimated 80,470 adults (61,700 men and 18,770 women) and 17,670 deaths (12,870 men and 4,800 women) in 2019 [1, 2]. For respective incidence and mortality rates, men are about four times higher than women globally. Besides, incidence rates in white men are double those of black men [3]. On the other hand, BLCA patients tend to older adults. Ninety percent of the patients are older than age 55 [4, 5]. Meanwhile, BLCA is third leading cancer and the fourth leading cause of cancer-related death in older men, and sixth and eighth in those of older women, separately [1, 6]. Finally, BLCA can be mainly divided into two subtypes based on the cancer cell infiltration: nonmuscle-invasive BLCA and muscle-invasive BLCA. The former has a high recurrence rate but less aggressive, while the latter has a relatively poor prognosis and is easier to metastasize [7–9]. It reports that BLCA has a high recurrence rate with over 50% of patients which at least have one recurrence within five years, and it possibly progresses to an aggressive, muscle-invasive, and even metastatic forms [10–12].

In clinical practice, the initial purpose of treatment is to slow down its development for early-stage BLCA. However, it is hard to achieve a better outcome based on the heterogeneous cancer feature [13] as well as their recurrent tendentiousness in time and location. At the same time, with the number of comorbidities increasing, it is complicated for clinicians too often making a challenging decision on how to choose effective treatment plans for an individual patient. It may take many resources in an aspect of humans, materials, and finances. Many authorities support the view of intensive surveillance and treatment for early-stage BLCA patients in the practice guidelines [14, 15]. It is implied that patients should prevent further progression or, at the very least, be able to detect recurrence early enough so that subsequent interventions are more successful and palatable. However, many cases may not readily satisfy a typical scenario in the published guidelines, thus leaving clinicians enough room to make a decision for the individual patient. And the essential evidence is poor and often due to expert experience and medical theory in terms of some view of these guidelines.

In the phase of staging and risk assessment, further imaging studies [16] will be completed to confirm the stage after patients have confirmed muscle invasion histology, such as computed tomography (CT) or magnetic resonance imaging (MRI). But both tests are often unable to reliably identify T2 from T3a, T3b, or even T4a, separately. For neoadjuvant and adjuvant therapy about muscle-invasive BLCA, the treatment plans are mainly from randomized trials, with lower methodological quality and suspicion of bias [17]. Meanwhile, there is still insufficient evidence for the routine use of adjuvant chemotherapy in early clinical stage (stage IA) practice [18]. High-risk patients may likely benefit most from adjuvant chemotherapy. And for further adjuvant chemotherapy, the clinical data is limited, so the evidence is not strong enough to guide treatment. As a result, clinicians lack quantitative and reliable estimates of competing for mortality risks when considering treatments, although there are guidelines for reference [19, 20]. At the same time, there is currently no efficient screening prognostic signature or method recommended for people in early-stage detection.

In this study, the purpose of a robust RWSR model is to find a prognosis signature that is strongly associated with clinical characters through quantitative analysis. And the signature possesses a potential prognostic value for patients with early-stage BLCA and may provide new information for research and treatment. Details are shown as follows. Firstly, the risk coefficient is calculated for each gene through the risk regression algorithm using the mRNA gene expression level and overall survival in the clinical dataset [21]. Genes with zero coefficients are excluded. Secondly, the risk probability is calculated for the remaining genes through a risk estimation algorithm using a gene expression value and risk coefficient. Finally, the risk probability is considered a parameter, and the risk coefficient is considered a dependent variable in the weighted least absolute deviation-smoothly clipped absolute deviation (WLAD-SCAD) algorithm. The genes of nonzero coefficients are identified to be candidate signature in both of GSE13507 and TCGA-BLCA, separately. The common genes between the two datasets construct the gene signature. After that, a series of statistical analyses are performed to validate how accurate, independent, and significant is the 17-gene signature individually.

Besides, there are 11 genes (PRKDC, FRY like transcription coactivator (FRYL), synaptopodin (SYNPO), Fc fragment of IgG receptor IIIb (FCGR3B), retention in endoplasmic reticulum sorting receptor 1 (RER1), CCDC73, ATPase H+/K+ transporting subunit alpha (ATP4A), contactin associated protein family member 4 (CNTNAP4), growth differentiation factor 7 (GDF7), PR/SET domain 14 (PRDM14), and EWS RNA binding protein 1 (EWSR1)) in the 17-gene signature that are validated to be a prognosis signature or strongly associated with other types of cancer already. The other six genes (GSG1 like (GSG1L), crumbs cell polarity complex component 1 (CRB1), XK-related 8 (XKR8), zinc finger protein 680 (ZNF680), zinc finger protein 284 (ZNF284), and zinc finger protein 780B (ZNF780B)) are not mentioned in the cancer research area until now. Among them, ZNF680, ZNF284, and ZNF780B are members of the zinc finger gene (ZNF) family which plays an essential role in the regulation of transcription [22]. And many members of the ZNF family are associated with cancer, including breast cancer, colorectal cancer, and gastric cancer [23–25]. Based on fundamental enrichment analysis, it demonstrated that the 17-gene signature significantly participated in immune-, cell cycle-, and transport-associated biological processes.

## 2. Materials and Methods

### 2.1. Data Collection

In this study, we download mRNA expression profile data and corresponding early-stage (I-III) clinical information of BLCA patients from GEO and TCGA, respectively. The gene expression data of the GEO dataset was calculated on Affymetrix U133 Plus 2.0 microarray platform and contained mRNA gene expression profile and clinical information of 256 patients from Chungbuk National University Hospital in GSE13507 Series (https://www.ncbi.nlm.nih.gov/geo/query/acc.cgi?acc=GSE13507). And another dataset was analyzed on the Illumina sequencing platform and contained mRNA gene expression profile and corresponding clinical information of 408 patients from TCGA (https://portal.gdc.cancer.gov/). Samples with more than 30 percent of zero in gene expression values are excluded. Characters with missing values in the clinical dataset are excluded, such as not available and unknown.

### 2.2. Risk-Weighted Sparse Regression (RWSR) Model for Screening Signature

A risk-weighted sparse regression model is proposed to screen the 17-gene signature, which represents the relationship between prognosis of early-stage BLCA patients and mRNA gene expression level. This model is performed using the R language, including three steps in total. In the first step, we obtain the risk coefficient matrix between the mRNA gene expression level and overall survival by [26] in Equation (1) and Equation (2) as follows:

(1) $$\begin{array}{c} R\left(t,X\right)=R_{0}\left(t\right)e^{\left(\beta_{1}X_{1}+\beta_{2}X_{2}+\cdots +\beta_{n}X_{n}\right)}, \end{array}$$

(2) $$\begin{array}{c} {HR}_{i}=\frac{R\left(t,X_{i}\right)}{R\left(t,X_{i}\right)}=\frac{R\left(t,X_{i}\right)e^{\beta^{\prime }X_{i}}}{R\left(t,X_{i}\right)e^{\beta^{\prime }X_{j}}}=e^{\left[\beta^{\prime }\left(X_{i}-X_{j}\right)\right]},\;i,j=1,2,\cdots ,n, \end{array}$$

where *X*_1_ represents the gene expression matrix, *R*(*t*, *X*) represents overall survival, and *β*_1_, *β*_2_, ⋯, *β*_*n*_ is the risk coefficient. the log-likelihood function is shown in Equation (3) as follows:

(3) $$\begin{array}{c} l\left(\beta \right)=logL\left(\beta \right)=\overset{N}{\underset{i=1}{\sum }}\left[\beta *X_{i}-\log\left(\underset{j=1}{\sum }e^{\left(\beta *X_{i}\right)}\right)\right]. \end{array}$$

Then, the risk coefficient is calculated according to the Newton-Raphson algorithm in Equation (4) as follows:

(4) $$\begin{array}{c} \beta^{\left(m+1\right)}=\beta^{\left(m\right)}-{\left(\frac{\partial^{2}l\left(\beta^{\left(m\right)}\right)}{\partial \beta \partial \beta^{\prime }}\right)}^{-1}*\frac{\partial l\left(\beta^{\left(m\right)}\right)}{\partial \beta }, \end{array}$$

where

(5) $$\begin{array}{c} \frac{\partial^{2}l\left(\beta \right)}{\partial \beta \partial \beta^{\prime }}=-\overset{n}{\underset{i=1}{\sum }}\delta_{i}\left(\frac{\sum_{j=1}^{n}e^{\beta^{\prime }X_{i}}{X_{i}}^{⨂2}}{\sum_{j=1}^{n}e^{\beta^{\prime }X_{i}}}-{\left(\frac{\sum_{j=1}^{n}e^{\beta^{\prime }X_{i}}X_{i}}{\sum_{j=1}^{n}e^{\beta^{\prime }X_{i}}}\right)}^{⨂2}\right), \end{array}$$

a new gene set is obtained based on the result of the Cox regression algorithm.

(6) $$\begin{array}{c} X_{i}=\left\{\begin{array}{c} X_{\text{risk},\;}{\text{HR}}_{i}>1\;,\beta_{i}>0,\\ 0,\;{\text{HR}}_{i}=1,\beta_{i}=0,\\ X_{\text{prot},}\text{ H}{\text{R}}_{i}<1,\beta_{i}<0. \end{array}\right. \end{array}$$

Referring to Equation (6), if HR_*i*_ < 1 and  *β*_*i*_ < 0, the corresponding genes are identified as candidate protective genes. If  HR_*i*_ > 1 and  *β*_*i*_ > 0, the relevant genes are defined as risky candidate genes. And if  HR_*i*_ = 1 and  *β*_*i*_ = 0, the relevant genes are a nonassociate factor.

In the second step, to obtain the risk probability matrix, we calculate the risk score and risk probability for each patient using the risk coefficient in Equation (7) and the gene expression value in Equation (8):

(7) $$\begin{array}{c} \text{rs}=\overset{n}{\underset{i=1}{\sum }}\left(X_{i}\;|\;{\text{HR}}_{i}\neq 1\&\beta_{i}=0\right)*\;\beta_{i}, \end{array}$$

(8) $$\begin{array}{c} \text{rp}=\frac{\left(X_{i}\;|\;{\text{HR}}_{i}\neq 1\&\beta_{i}=0\right)*\;\beta_{i}}{\sum_{i=1}^{n}\left(X_{i}\;|\;{\text{HR}}_{i}\neq 1\&\beta_{i}=0\right)*\;\beta_{i}}, \end{array}$$

where *n* represents the number of genes and *X*_*i*_ represents the *i*^th^ gene expression value. BLCA patients were separated into high-risk and low-risk groups by the median value of the risk score as a cutoff value.

In the last step, to further screen the gene signature, [27] proposed a sparse linear regression model (WLAD-SCAD), considering rp as a parameter of *X*_*i*_, given by Equation (9) as follows:

(9) $$\begin{array}{c} Q_{n\omega }\left(\gamma_{n}\right)=\overset{n}{\underset{i=1}{\sum }}\omega_{i}\left|{\text{rs}}_{i}-\text{rp}*X_{i}^{T}\gamma_{n}\right|+n\overset{p_{n}}{\underset{j=1}{\sum }}p_{\lambda }\left(\left|\gamma_{nj}\right|\right), \end{array}$$

where *Q*_*nω*_(*γ*_*n*_) is the objective function, *p*_*λ*(.)_ is SCAD penalty function, *X*_*i*_ is the gene expression value, rs_*i*_ is the overall survival, and *γ* = (*γ*_1_, *γ*_2_, ⋯*γ*_*p*_)^*T*^ represents the regression coefficient.

In order to calculate the objective function, [28] proposed an efficient weighted method, the process is shown as follows:

Firstly, in order to compress the dataset into arranging (0,1), a transformation of *X*_*i*_ is given by Equation (10) as follows:

(10) $$\begin{array}{c} {\overset{ˇ}{x}}_{j}^{i}=\frac{x_{j}^{i}\;-\underset{1\leq j\leq p_{n}}{\min}\left\{x_{j}^{i}\right\}}{\underset{1\leq j\leq p_{n}}{\max}\left\{x_{j}^{i}\right\}\;-\underset{1\leq j\leq p_{n}}{\min}\left\{x_{j}^{i}\right\}},\;i=1,2,\cdots ,p_{n}, \end{array}$$

where *x*_*j*_^*i*^ represents the *i*^th^ row, *j*^th^ column element of matrix X and ${\overset{ˇ}{x}}_{j}^{i}$ represents the *i*^th^ row, *j*^th^ column element of the matrix $\overset{ˇ}{X}$. Secondly, the Euclidean distance is used to calculate the center distance given by Equation (11) as follows:

(11) $$\begin{array}{c} d_{i}=\left\|{\overset{ˇ}{x}}^{i}-\overset{ˇ}{m}\right\|,i=1,2,\cdots ,n, \end{array}$$

where ${\overset{ˇ}{x}}^{i}$ is the *i*^th^ row, $\overset{ˇ}{m}$ is the median vector of ${\overset{ˇ}{x}}^{i}$, and ‖.‖ represents the Euclidean distance. To obtain *d*^*i*^, *i* = 1, 2, ⋯, *n*, we order *d*_*i*_ in a decreasing sequence. In the end, a subset is constructed in Equation (12) as follows:

(12) $$\begin{array}{c} X_{S}={\overset{ˇ}{X}}_{\left(l*p_{n}\right)}={\left({\overset{ˇ}{x}}^{1},\;\cdots ,\;{\overset{ˇ}{x}}^{l}\right)}^{T}, \end{array}$$

where center distance of ${\overset{ˇ}{x}}^{i}$ is *d*^*i*^, (1 ≤ *i* ≤ *l* = 0.6∗*n*). Finally, the weight function is calculated by Equation (13), which can not only avoid heavy calculation burden but also improve the robustness of estimation, shown as follows:

(13) $$\begin{array}{c} \omega_{i}=\sqrt{\underset{j}{\min}h_{j}/h_{i}}=\sqrt{\underset{j}{\min}\left(x^{j}{\left(X_{S}^{T}X_{S}\right)}^{-1}x^{jT}\right)/x^{i}{\left(X_{S}^{T}X_{S}\right)}^{-1}x^{iT}}. \end{array}$$

Obviously, the weight is inversely proportional to the subset size of the center distance, which can greatly reduce the impact of outliers on regression, and wlad has better robustness than other methods. In order to calculate Equation (9), the penalty Equation (14) is local linear approximation through [29]:

(14) $$\begin{array}{c} {\left[p_{\lambda n}\left|\gamma_{nj}\right|\right]}^{\prime }=p_{\lambda }^{\prime }\left(\left|\gamma_{nj}\right|\right)\mathrm{sgn}\left(\gamma_{nj}\right)\approx \left\{p_{\lambda_{n}}^{\prime }\left(\left|\gamma_{n0j}\right|\right)/\left|\gamma_{n0j}\right|\right\}\gamma_{nj}, \end{array}$$

where *γ*_*nj*_ ≠ 0, and

(15) $$\begin{array}{c} \mathrm{sgn}\left(x\right)=\left\{\begin{array}{c} 1,\;x>0,\\ 0,\;x=0,\\ -1,\;x<0, \end{array}\right. \end{array}$$

then,

(16) $$\begin{array}{c} p_{\lambda_{n}\left(\left|\gamma_{nj}\right|\right)}\approx p_{\lambda_{n}\left(\left|\gamma_{n0j}\right|\right)}+\frac{1}{2}\left\{p_{\lambda_{n}}^{\prime }\left(\frac{\left(\left|\gamma_{n0j}\right|\right)\;}{\left(\left|\gamma_{n0j}\right|\right)}\right)/\right\}\left(\gamma_{nj}^{2}-\gamma_{n0j}^{2}\right),\;\gamma_{nj}\approx \gamma_{n0j}, \end{array}$$

*γ*_*n*_ is calculated based on

(17) $$\begin{array}{c} \gamma_{n}^{\left(k+1\right)}=\arg\underset{\gamma }{\min}\left\{\overset{n}{\underset{i=1}{\sum }}\left|{rs}_{i}^{*}-{rp}_{i}*{x_{i}^{*}}^{T}\gamma_{n}\right|+n*\overset{p_{n}}{\underset{j=1}{\sum }}\frac{p_{\lambda_{n}}^{\prime }\left(\left|\gamma_{nj}^{\left(k\right)}\right|\right)}{2\left|\gamma_{nj}^{\left(k\right)}\right|}\gamma_{nj}^{2}\right\},\;\;k=0,1,2,\cdots ,m. \end{array}$$

In order to improve reliability, only interaction genes between the two datasets are identified to construct the prognostic signature.

### 2.3. Prognosis Model

After screening the signature through the RSWR model, the multiple Cox proportional hazard regression model is used to estimate whether the signature could be an independent prognostic factor for patient survival. A multigene-based prognostic risk score is constructed in Equation (18) as follows:

(18) $$\begin{array}{c} \text{risk scor}{\text{e}}_{p}=\overset{n}{\underset{i=1}{\sum }}\;X_{i}*\;\gamma_{i},\;\;p=1,2,\cdots ,m, \end{array}$$

where *n* is the number of prognostic genes, *p* is the number of patients, *X*_*i*_ represents the expression level of gene *i*, and *γ*_*i*_ the regression coefficients from the multivariate Cox regression model. A risk score is considered a prognostic index. Taking the median risk score as a cutoff value, patients from TCGA-BLCA and GES13507 are divided into high-risk and low-risk groups. The univariate and multivariate Cox regression analyses are applied to evaluate the prognostic role of the risk score, along with age, gender, grade, and TNM stage.

### 2.4. Functional Enrichment Analysis

The functional enrichment analysis of Gene Ontology (GO) and Kyoto encyclopedia of genes and genomes (KEGG) is conducted using the hypergeometric distribution method to identify significantly enriched biological themes including GO terms and KEGG pathways. GO functional terms limited in the “Biological Process” and KEGG pathways with a *P* value < 0.01 are considered significant. Four pathway databases and one GO terms are downloaded from Explore the Molecular Signatures Database (MSigDB) for doing enrichment analysis, including GO-BP database, KEGG pathway database, Reactome pathway database, Pathway Interaction Database (PID), and BioCarta pathway database.

### 2.5. Statistical Analysis

To predict the differences between the two risk BLCA patient groups based on survival time, we use the Kaplan-Meier method and calculate the log-rank value to identify the statistical significance between groups. Multivariable Cox regression analysis and stratification analysis are used to estimate the independence of the risk score with other clinical factors [30]. Time-dependent receiver operational feature (ROC) curve analyses are made to evaluate the predictive capacity of the model [31]. And AUC is compared to judge the prognostic performance of Cox analysis, which is the area under the ROC curve with a significance of *P* < 0.05. In addition, comprehensive survival analysis is also implemented to analyze the relationship between the different clinical characters (stage, grade, and age) and the prognosis model. The *P* value < 0.05 is used as a cutoff during the prognostic analysis.

## 3. Result

### 3.1. Prognostic Signature Generation

**Figure 1:**
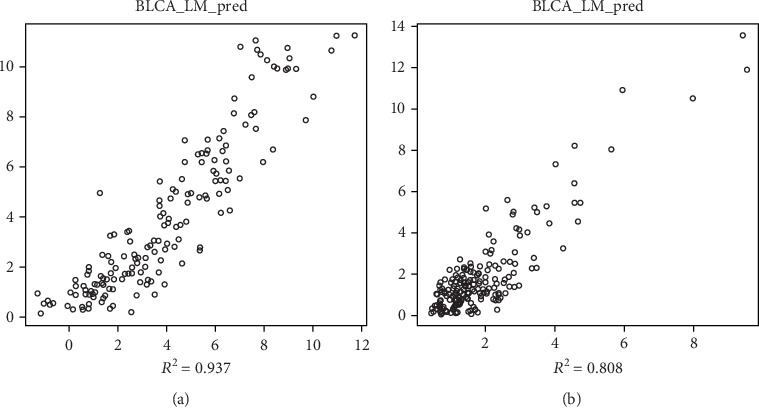
The RWSR model for GSE13507 (a) and TCGA (b). The *x*-axis represents the predicting survival value, and *y*-axis represents the actual value.

The novel model RWSR is used to identify the gene signature, which is significantly associated with the overall survival of BLCA patients. In the first step, with *P* < 0.01 and hazard ratio (HR) <1 as the cutoff value, 617 genes and 1761 genes are selected to be candidate protective genes in GSE13507 and TCGA individually. With *P* < 0.01 and HR > 1 as the cutoff value, 1,399 genes and 2096 genes are selected to be risky candidate genes in GSE13507 and TCGA, separately. It is named com_prot that the intersection of two candidate protective gene sets from both initial datasets, with 268 genes. Similarly, it is called com_risk for standard risk gene sets with 189 genes in total. Based on the second step, the risk score and risk probability are obtained individually. Followed by the last step, the genes with a nonzero coefficient are identified, 106 genes and 403 genes have remained in the gene set of com_pret_geo and com_pret_tcga. Meanwhile, 327 genes and 496 genes have remained in the gene set of com_risk_geo and com_risk_tcga. Only common genes on the two datasets are considered candidate signature reliability. As a result, a 17-gene signature is identified, which is strongly correlated with overall survival depending on two independent datasets, including 5 protective genes and 12 risky genes, separately. In order to validate the fitness of the novel model directly, a point plot presents the relationship between the predicted risk score value and actual value, as shown in Figure 1. In Figure 1, there is a value *R*^2^ shown in Figure 1, which is the determination coefficient, also known as the goodness of fit. The arrangement is between 0 and 1; the larger the value is, the higher the fitting degree between the regression model and the actual data is. It is evident that fitness is better in which the value of R2 is 0.937 in GES13507 (Figure 1(a)) and 0.808 in TCGA-BLCA (Figure 1(b)), separately. The points in Figure 1 are distributed near the diagonal of *y* = *x*. The general information of the 17 genes is displayed in Table 1. The prognostic analysis information of the 17 genes with overall survival of early-stage BLCA patients in both datasets is shown in Table 2.

### 3.2. 17-Gene Prognostic Signature Validation

**Figure 2:**
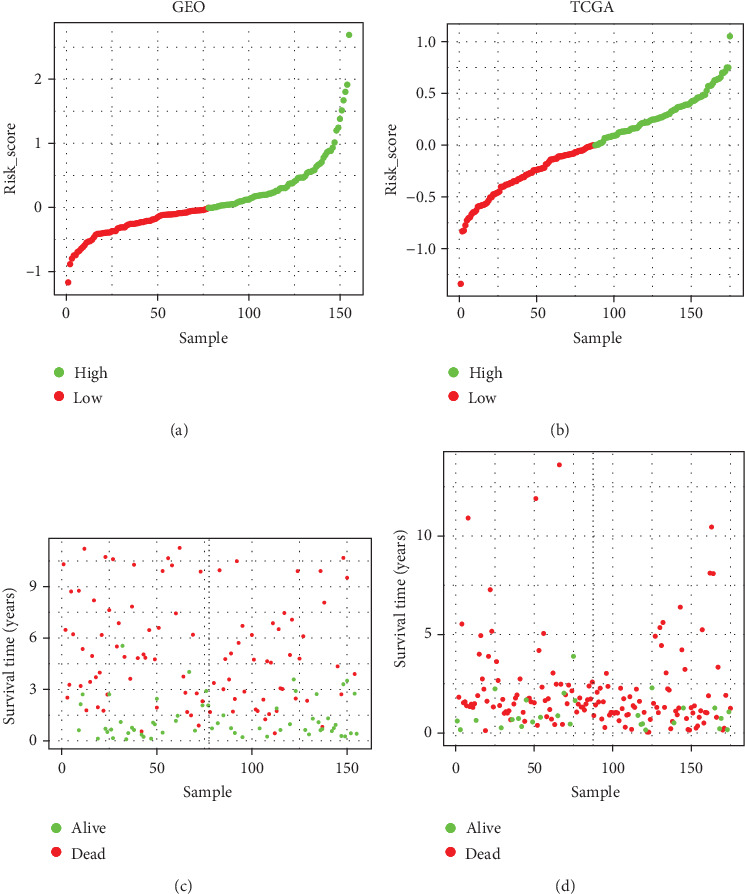
Risk-score analysis of early-stage BLCA patients in two datasets. For each dataset, distribution about the risk score and the samples' survival status is displayed based on the high-risk group and the low-risk group individually. (a) Distribution of the risk score in GEO. (b) Distribution of the risk score in TCGA. (c) Distribution of survival status in GEO. (d) Distribution of survival status in TCGA.

**Figure 3:**
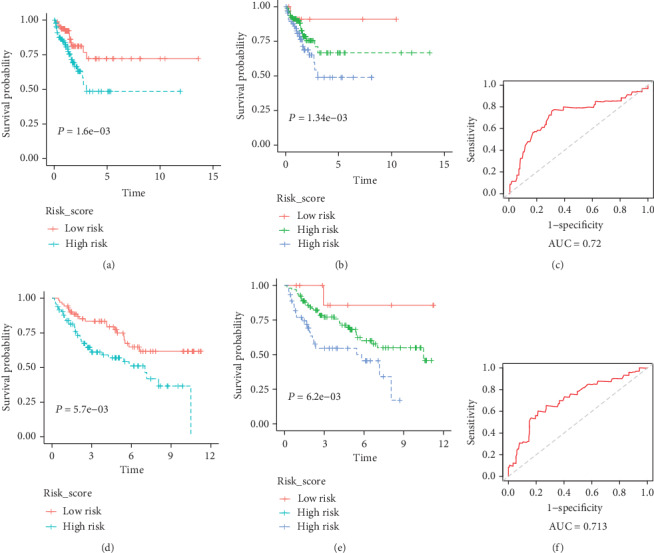
Kaplan-Meier and ROC curves of the 17-gene signature. The Kaplan-Meier curve of the signature in two risk groups (a), three risk groups (b), and ROC curve (c) of GEO. The Kaplan-Meier curve of the signature in two risk groups (d), three risk groups (e), and ROC curve (f) of TCGA. Patients with high-risk scores represent poor outcomes in terms of overall survival.

Based on the risk coefficients, a risk score is built up for signature. To the assess overall survival, a prognostic model is constructed. The patients are separated into the high-risk group and the low-risk group by using the median risk score as a cutoff point. In Figure 2, the risk score distribution and the patients' survival status in two datasets are displayed, ranked based on the risk score values for the 17-gene signature. It is obvious that the patients in the high-risk group has a shorter overall survival than those in the low-risk group (GSE13507: HR = 2.2129, 95%CI = (1.14 − 4.296), *P* = 1.6*E* − 03; TCGA: HR = 1.13094, 95%CI = (1.051 − 1.217), *P* = 0.0057) as shown in Figures 3(a) and 3(d). Then, we group patients into three parts and predict the survival difference, including high-risk, median-risk, and low-risk groups based on the risk score. The results show that patients with a higher risk score have a worse survival (GSE13507: *P* = 2.67*E* − 4; TCGA: *P* = 2*E* − 3) as shown in Figures 3(b) and 3(e). According to these results, the risk score can be considered a prognostic factor. The corresponding ROC curves present the accuracy (AUC) of th e17-gene signature up to values of 0.74 and 0.737 in GSE13507 and TCGA, respectively, as shown in Figures 3(c) and 3(f), which means the model has an effective performance for overall survival assessment.

**Figure 4:**
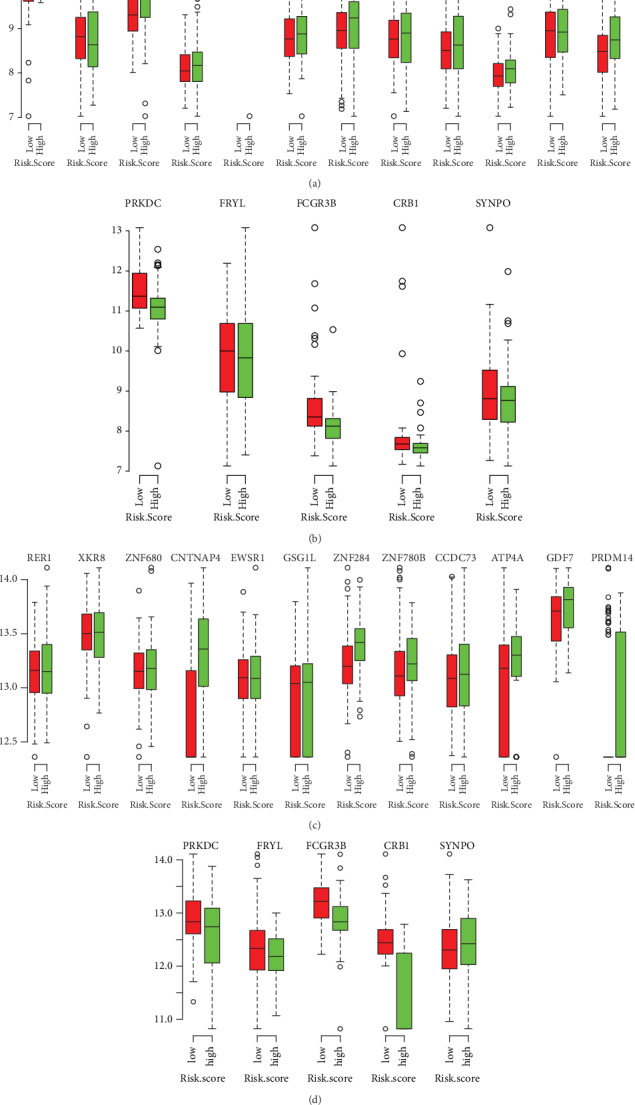
Box plot visualization of the mRNA gene expression level into risk groups in two datasets. (a) Risky genes in GEO. (b) Protective gene in GEO. (c) Risky genes in TCGA. (d) Protective gene in TCGA.

Among the 17-gene signature, 12 genes are associated with high risk (RER1, ZNF284, ZNF780B, XKR8, CCDC73, ATP4A, ZNF680, CNTNAP4, GDF7, PRDM14, EWSR1, and GSG1L; HR > 1) and five genes appear to be protective (PRKDC, SYNPO, FRYL, FCGR3B, and CRB1; HR < 1). We examine the expression level of the prognostic genes according to the comparison of the differences between high risk and low risk. It is evident that patients with high-risk scores prefer expressing risky genes, while patients with the low-risk group prefer expressing protective genes; the corresponding boxplot is shown in Figure 4.

### 3.3. The 17-Gene Prognostic Signature Is Independent of Other Clinicopathological Factors

We adopt the stepwise Cox regression analysis to estimate the impact of the 17-gene signature as an independent prognostic feature for patient survival. Covariates contain the gene signature and clinicopathological characters, including gender, age, stage, grade, smoking, invasiveness, time, and event status. The result confirms the independence of the estimate skills of the 17-gene signature comparing clinicopathological characters with overall survival of early-stage BLCA patients among the two datasets (GSE13507: HR = 4.02, 95%CI = 4.99 − 8.04, *P* = 2.46*E* − 04; TCGA: HR = 3.11, 95%CI = 3.09 − 4.24, *P* = 8.42*E* − 04) as shown in Table 3.

### 3.4. Stratification Analysis

**Figure 5:**
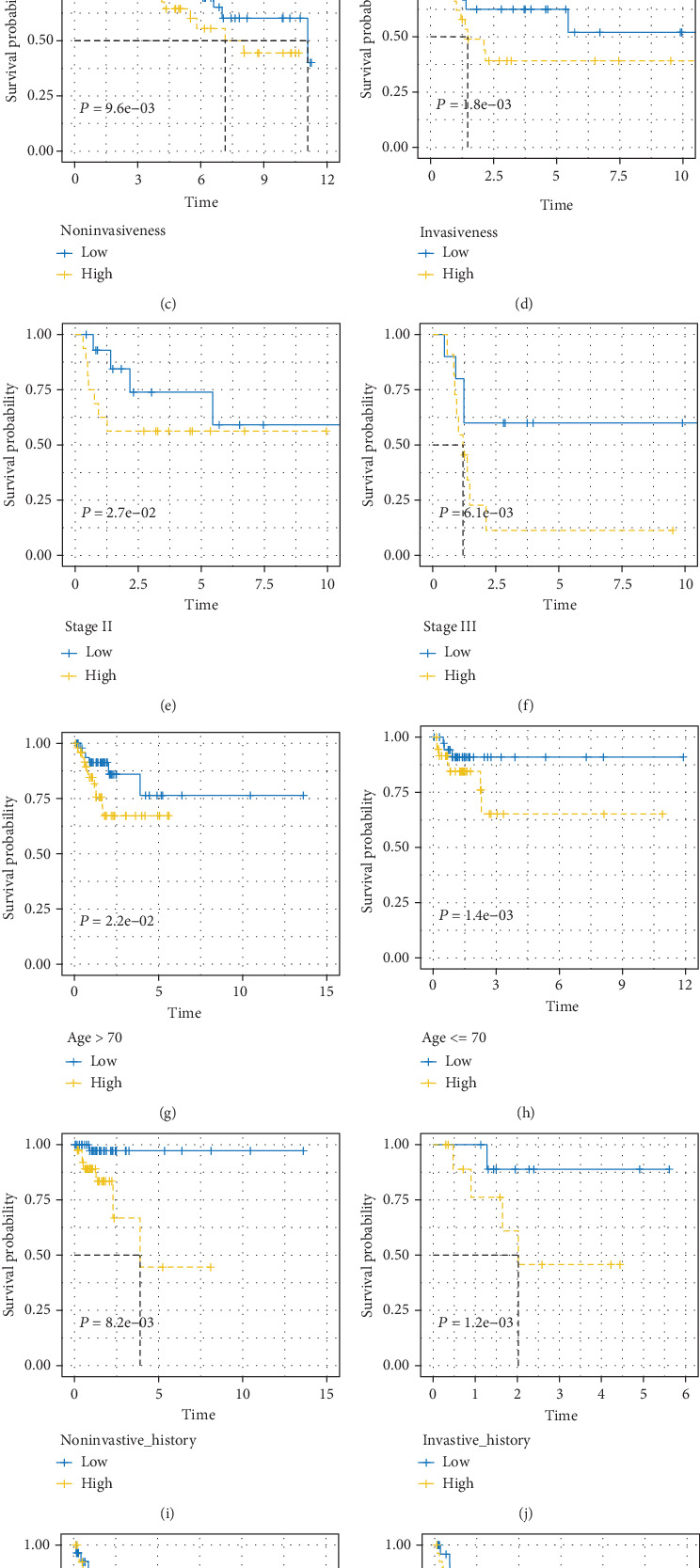
Kaplan-Meier analysis of overall survival stratified by age, stage, and invasiveness in GEO and TCGA. For GEO dataset, (a) older than 70, (b) younger or equal than 70, (c) noninvasiveness, (d) invasiveness, (e) stage II, and (f) stage III. For TCGA dataset, (g) older than 70, (h) younger or equal than 70, (i) noninvasiveness, (j) invasiveness, (k) stage II, and (l) stage III.

Some clinicopathological characters are also considered independent prognostic characters during multivariate Cox regression analysis. In order to assess the predictive ability of the 17-gene signature in the same clinical character subgroup, a stratification analysis is adopted in this study. Patients are manually stratified due to clinical characters, such as age (<=70/>70), gender (male/female), stage (0-III), and invasiveness (yes/no). The result shows that the 17-gene signature could divide patients with the same characters into high-risk and low-risk groups, separately. Patients with low-risk scores have a longer overall survival, and vice versa, which is shown in Figure 5.

### 3.5. Survival Prediction by Stage and 17-Gene Signature Combination

**Figure 6:**
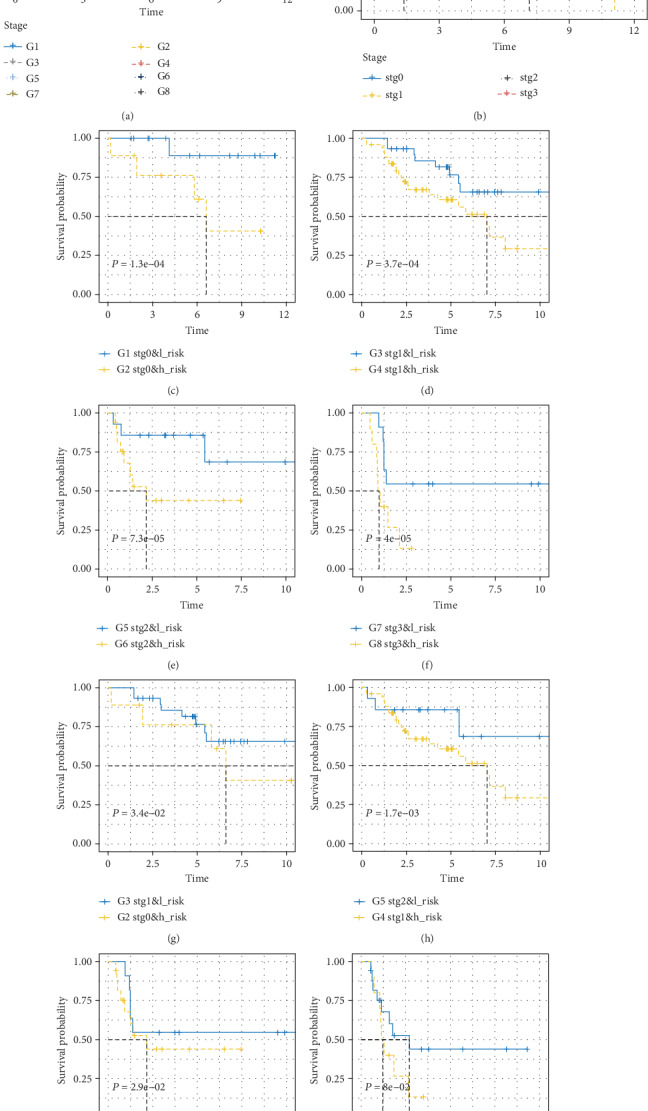
Kaplan-Meier analysis of overall survival grouped by stage and risk score combination. (a) Eight groups by stage and risk score. (b) Four stages. (c) Group 1 (stage 0 and low risk) and Group 2 (stage 0 and high risk). (d) Group 3 (stage I and low risk) and Group 4 (stage I and high risk). (e) Group 5 (stage II and low risk) and Group 6 (stage II and high risk). (f) Group 7 (stage III and low risk) and Group 8 (stage III and high risk). (g) Group 2 and Group 3. (h) Group 4 and Group 5. (i) Group 6 and Group 7. (j) Group 6 and Group 8.

It is proved that the tumor stage, as an emphasis clinical character, has a significant survival predictive value in clinical management. In this study, the stage and the risk score are confirmed as independent prognostic factors in two independent datasets individually. Therefore, a further prognostic model is constructed for survival assessment, trying to integrate the character of stage and 17-gene signature. Based on the stage status and the risk score, patients are divided into eight independent groups: Group 1 (stage 0 and low risk), Group 2 (stage 0 and high risk), Group 3 (stage I and low risk), Group 4 (stage I and high risk), Group 5 (stage II and low risk), Group 6 (stage II and high risk), Group 7 (stage III and low risk), and Group 8 (stage III and high risk), which are shown in Figure 6(a). Patients are all classified into high-risk and low-risk groups under stages 0 to III in both datasets. And in general, patients in the high-risk group have a poor prognosis. According to the result demonstrated in Figure 6, the patients in the high-risk group have worse outcomes than those in the low-risk group among the same stage. It means patients in Groups 2, 4, 6, and 8 are worse than in Groups 1, 3, 5, and 7 individually. However, there are no significant changes in the overall survival among patients in Group 2 and Group 3 as shown in Figure 6(g). Meanwhile, the overall survival in patients of Group 6 and Group 7 is nearly the same as shown in Figure 6(i). These results suggest that patients with a high-risk score in stage II might have similar prognosis as those with a low-risk score in stage III, suggesting that intravesical therapy and neoadjuvant chemotherapy should also be used in patients diagnosed as stage II with a high-risk score.

Among the eight groups, Group 1 demonstrates the best prognosis result obviously; on the contrary, Group 8 displays the worst. In the future clinical practice, patients can be divided into eight groups due to stage information and risk scores to estimate treatment outcomes through the model proposed in this study.

### 3.6. Signature Enrichment Analysis

To obtain more insights into the functional roles of the 17-gene signature in BLCA, we performed enrichment analysis for the signature to investigate the associated biological processes and pathways. A *P* value < 0.002 is considered statistically significant as shown in Table 4. And according to the ascending order of *P* values, the top five GO-BP terms are GO cell fate commitment, GO negative regulation of the fibroblast growth factor receptor signaling pathway, GO negative regulation of cellular senescence, GO lymphoid progenitor cell differentiation, and GO sodium ion export. The other results of the GO-BP term are shown in Supplementary Table S1.

## 4. Discussion

Due to cancer heterogeneous and complex molecular mechanisms, BLCA remains one of the most commen malignancies in the world. BLCA patients still face a crisis of mortality. And BLCA clinicians are hard to make an efficient management decision as they lack of reliable assessment of prognosis. In this study, a novel prognostic signature is identified and validated through RNA-seq data and plenty of clinical data from early-stage BLCA patients in two independent datasets. The discovered gene signature can discriminate early-stage BLCA patients between high and low risk in poor prognosis. This feature may improve BLCA surveillance in clinical treatment and may be an emphasis step in making treatment decisions for early-stage BLCA patients.

### 4.1. RWSR Model for Screening the Prognostic Signature

In order to screen the gene signature effectively, an RWSR model is proposed to describe the relationship between the prognosis of early-stage BLCA patients and the mRNA gene expression levels. This model is mainly based on a sparse linear regression algorithm. We consider the risk possibility of each gene and the Euclidean distance of the prognosis index as restricted parameters of the linear regression algorithm [32–37], which is the difference between existing methods and RWSR. In order to improve reliability, only interaction genes between the two datasets are identified to construct the prognostic signature. An index for validation of the model is the goodness of fit, which is larger than 0.8 (range [0, 1]) in two linear models. The larger the value is, the higher the fitting of the model. The other index is the *P* value, which represents the statistical significance of the model. In this study, two models are significant with a *P* value less than 2.2*E*-16 and a value of *R* square more than 0.8 in Figure 1, respectively.

### 4.2. Prognosis Model through the 17-Gene Signature

A prognosis model is established by the risk score and multivariate Cox analysis model for predicting BLCA outcomes. The corresponding ROC curves present the accuracy (AUC) of the signature up to values of 0.74 and 0.737 in GSE13507 and TCGA, respectively, suggesting that the 17-gene signature has good survival prediction performance in Figure 3. Our study found that high expression levels of the gene signature are associated with poor prognosis in early-stage BLCA patients. We demonstrated that the 17-gene signature and the risk score are independent prognostic factors superior to traditional clinicopathological factors and verified their survival prediction ability in GEO, shown as Table 3. Thus, it is proved that grouping BLCA patients into the high-risk and low-risk groups by the 17-gene-based risk scoring model can be considered early prevention or detection of BLCA recurrence in high-risk patients. The gene signature is derived from a common gene set, and different kinds of survival analysis are adopted to validate the possibility and accuracy of prediction ability for prognostic and detection in early-stage BLCA.

### 4.3. Prognostic Signature for BLCA

As a result of this study, the 17-gene signature is identified to be an independent prognosis factor. The general information of the signature is shown in Table 1 and Table 2. Among it, ATP4A, which encodes protein as a family member of P-type cation-transporting ATPases, catalyzes the hydrolysis of ATP coupled with the exchange of H(+) and K(+) ions across the plasma membrane, being responsible for acid production in the stomach [38]. Meanwhile, ATP4A and ATP4B downregulation involve DNA methylation and methylated ATP4B DNA in the plasma are potential biomarkers for gastric cancer [39, 40]. CNTNAP4 which encodes a member of the neurexin protein family, which function in the vertebrate nervous system, is considered cell adhesion molecules and receptors [41]. Meanwhile, in breast cancer patients, 16q deletion is associated with survival, molecular subtypes, mRNA expression, and germline haplotypes, and the cell recognition gene CNTNAP4 is included [42]. EWSR1 encodes a multifunctional protein that is involved in various cellular processes, including gene expression, cell signaling, and RNA processing and transport [43]. Mutations in this gene are known to cause Ewing sarcoma as well as neuroectodermal and various other tumors [44, 45]. Alternative splicing of this gene results in multiple transcript variants. The fusion of short fragments between EWSR1 and FLI1, contributing to the oncogenic gene to construct and maintain expression programs, is enough to recapitulate BAF complex retargeting and EWS-FLI1 activities [46]. GDF7 encodes a secreted ligand of the TGF-beta superfamily of proteins, which binds various TGF-beta receptors leading to recruitment and activation of SMAD family transcription factors that regulate gene expression [47]. A mutation in this gene may be associated with increased risk for Barrett's esophagus and esophageal adenocarcinoma [48, 49]. PRDM14 encodes a protein that may possess histone methyltransferase activity. It plays a critical role in cell pluripotency by suppressing the expression of differentiation marker genes. Expression of this gene can reduce the tumor size and distant metastasis of these cells in nude mice, which may be an effective and radical therapy for solid cancers, such as pancreatic cancer, testicular cancer, and breast cancer [50–52]. Its related pathways are developmental biology and transcriptional regulation of pluripotent stem cells [53]. An important paralog is PRDM6. CCDC73 is associated with ovarian cancer [54], hepatocellular carcinoma [55], and endometrial cancer [56]. RER1 enhances carcinogenesis and the stemness of pancreatic cancer under the hypoxic environment [57], which is associated with hepatocellular carcinoma [58]. The protein encoded by FCGR3B is a low-affinity receptor for the Fc region of gamma immunoglobulins (IgG), which function is to capture immune complexes in the peripheral circulation. FCGR3B is associated with innate immune system-related disease, including neonatal alloimmune neutropenia and eosinophilic granulomatosis with polyangiitis [59]. The function of FCGR3B is associated with the innate immune system, renal cell carcinoma, and antiglomerular basement membrane antibody disease (anti-GBM disease) [60]. Meanwhile, FCGR3B may be a helpful prognostic tool for patients with metastatic carcinoma [61]. The research indicates that FRYL is a direct target of miR-1205 through experience and calculation methods. Meanwhile, miR-1205 regulates the proliferation and migration of prostate epithelial cells, and loss of miR-1205 promotes a tumorigenic phenotype in prostate cancer. Consequently, strategies to increase miR-1205 or target FRYL may have therapeutic potential in androgen-independent prostate cancer [62]. A previous study has implicated that downregulation of PRKDC-sensitized MCF-7 cells to chemodrugs in vitro, which is a potential prognostic and predictive marker of response to adjuvant chemotherapy in breast cancer patients [63]. It is proved that PRKDC expression is significantly increased in breast cancer tissue samples compared with NATs and is correlated with reduced overall and progression-free survival in high-grade glioma patients [64]. Downregulation of PRKDC reduces colorectal cancer cell proliferation/survival and induces apoptosis partially through inhibiting AKT activation in colorectal cancer cells. Meanwhile, PRKDC has no relationship with tumor growth but is associated with OS in colorectal cancer patients [65]. Spinal miRNA-124 regulates SYNPO and nociception in an animal model of bone cancer pain [66], so SYNPO is upregulated in bone cancer.

However, there are no any reports or laboratory data on the relationship between cancer and the following genes: GSG1L, CRB1, XKR8, ZNF680, ZNF284, and ZNF780B, which is part of the 17-gene signature. Among them, according to the latest report, GSG1L is associated with the plasma concentration of methadone, which can predict treatment responses and methadone-related deaths for individuals. Methadone maintenance treatment is commonly used for controlling opioid dependence, preventing withdrawal symptoms, and improving the quality of life of heroin-dependent patients [67]. XKR8 can be considered a specific signal for engulfment. CRB1 maintains cell polarization and adhesion. And mutation of CRB1 is correlated with a severe form of retinitis pigmentosa and with Leber congenital amaurosis [68]. But its homolog protein CRB3 may mediate the extracellular signal transduction in clear cell renal cell carcinoma development via an intracellular signal, such as Hippo signal [69]. ZNF680, ZNF284, and ZNF780B are all members of the zinc finger gene (ZNF) family which is one of the vertebrate transcription factors (TF). But the ZNF family is a notable exception that novel ZNF gene types have arisen, duplicated, and diverged independently throughout evolution to yield many lineage-specific TF genes, which makes identification of ZNF complicated [70]. And many members of the ZNF family are associated with cancer, including breast cancer, colorectal cancer, hepatocellular carcinoma, and lung cancer [71–74].

It is noteworthy that, in predicting survival status, the AUC value of the 17-gene signature is more significant than 0.6 in two datasets, respectively. This means a combination of 17 genes can be considered a new prognosis indicator for BLCA patients. Besides, the 17-gene signature represents the powerful ability to divide BLCA patients into a high- and low-group using stratification analysis. As a result, it could be a vital method considered in providing better prescriptions and improving prognosis.

### 4.4. Enrichment Analysis for the 17-Gene Signature

In the functional enrichment analysis, 24 pathways are significantly enriched among signature genes, including cell cycle, signaling pathway, and transport pathway, which is shown in Table 4. And 142 GO terms of biological process are enriched considerably among the 17-gene signature in Supplementary Table S1., including the signaling pathway, immune response, cell development/aging, regulation of the immune process, and transport. Taken together, it demonstrated that the signature significantly participated in immune-, cell cycle-, and transport-associated biological processes, which are cancer-related biological activities [75].

Nowadays, the tumor staging system is still the most essential tool of survival prediction and treatment decisions for BLCA patients. Despite having a large clinical value, it is not enough to guide management of its ability on prognosis and prediction. In particular, the present staging system is far from estimate survival at an individual, because 50% of early-stage patients will develop to be recurrence disease [76]. This is directly associated with the decision on intravesical instillations after transurethral resection of bladder tumor (TURBT) of early-stage patients. So, it is helpful for the clinician to screen suitable candidate patients of adjuvant chemotherapy that confirmation poor prognosis on the early-stage patient. Meanwhile, it is possible to help patients stratify by further development of genomic features in clinical practice.

### 4.5. Validation of the 17-Gene Signature for BLCA

In stratification analysis, the 17-gene signature could dedicate the prognosis value of patients in stages 0-III. Furthermore, it could separate patients whose survival prospects are significantly different in the same stage into high- and low-risk group, which means the signature may improve the accuracy of survival prediction in Figure 5. We can also see that age, stage, and invasiveness could separate patients into high- and low-risk groups in two independent datasets individually. And the *P* value is less than 0.04. Additionally, a prognosis model, estimating survival, is constructed to integrate characters of the 17-gene signature. We find that survival of patients in Group 3 are better than those in Group 2, Group 5 are better than Group 4, and Group 7 are better than Group 6 in Figure 6, which means survival of parts with high-stage patients have longer survival time than those with low-stage patients. These results demonstrate that the patients with a high risk score in stage 0 should received the same close monitoring with stage I patients with a low risk score, patients with high risk score in stage I should accept the same treatment as those with a low risk score in stage II, and the patients with a high risk score in stage II should take out the same therapy as those with a low risk score in stage III. The discovery may help clinicians to select high-risk patients except transurethral resection (TUR) doing adjuvant chemotherapy.

Importantly, it reveals that stage and overall survival are significantly correlated no matter which univariant or multivariant Cox regression model is, which is shown in Table 3. We can also see that the value of HR in sex, age, stage, grade, and risk score is more than 1 for both datasets. In addition, the overall survival of some patients in low stage I is worse than that in high stage, it may be that the reason is the extra reexam time needed by a clinician in order to decide the second step diagnosis plan after initial treatment. But it could be more efficient and cheaper if patients are grouped into high- and low-risk at the very beginning according to the prognosis model constructed in this study.

The findings in this study may have significant clinical value for early-stage BLCA patients. Significantly, there are still a few limitations existing. Firstly, the clinical information is incomplete, so some records with missing information cannot be used in the study. Secondly, it should be more comprehensive in the future research. The microenvironment, different kinds of data and more analysis methods would be considered into the next step, such as the immune environment, lncRNA, and optimized model.

## 5. Conclusion

A robust 17-gene signature is identified to predict the prognosis of early-stage BLCA patients. The results show that the 17-gene signature is a powerful prediction factor for the overall survival of early-stage BLCA patients. In addition, this signature is an independent factor for prognosis. There is no relationship with any other clinical characters, such as stage status. Finally, a prognostic model was proposed combining the gene feature of the 17 genes and stage information of BLCA patients. This study might help prognosis and treatment individually more accurately, especially for high-risk BLCA patients. So, it has clinical practice significance.

In the future, more mRNA datasets could be adopted to identify signature, which could reduce the range of signature and improve the accuracy. On the other hand, multiple omics data analysis could be tried to improve our result.

## Figures and Tables

**Table 1:** General information of the 17 genes for constructing the prognostic signature.

Gene_id	Symbol	Gene_type	Chromosome	Strand	Gene start–Gene end
ENSG00000105675.8	ATP4A	Protein_coding	19	—	36,040,945-36,054,560
ENSG00000186714.12	CCDC73	Protein_coding	11	—	32,623,626-32,816,204
ENSG00000152910.18	CNTNAP4	Protein_coding	16	+	76,311,176-76,593,135
ENSG00000134376.15	CRB1	Protein_coding	1	+	197,170,592-197,447,585
ENSG00000182944.17	EWSR1	Protein_coding	22	+	29,663,998-29,696,515
ENSG00000162747.10	FCGR3B	Protein_coding	1	—	161,592,986-161,601,753
ENSG00000075539.14	FRYL	Protein_coding	4	—	48,499,378-48,782,339
ENSG00000143869.6	GDF7	Protein_coding	2	+	20,866,424-20,873,418
ENSG00000169181.12	GSG1L	Protein_coding	16	—	15637866-15842235
ENSG00000147596.3	PRDM14	Protein_coding	8	—	70,963,886-70,983,928
ENSG00000253729.7	PRKDC	Protein_coding	16	+	15637866-15842235
ENSG00000157916.19	RER1	Protein_coding	4	—	155074109-155086371
ENSG00000171992.12	SYNPO	Protein_coding	5	+	149,980,642-150,038,792
ENSG00000158156.7	XKR8	Protein_coding	1	+	28,285,973-28,294,607
ENSG00000186026.6	ZNF284	Protein_coding	19	+	44,576,297-44,593,766
ENSG00000173041.11	ZNF680	Protein_coding	7	—	63,980,255-64,023,505
ENSG00000128000.15	ZNF780B	Protein_coding	19	—	40,534,167-40,562,116

**Table 2:** Univariate Cox regression analysis of 17 genes and overall survival of BLCA in two databases.

Gene name	GEO		TCGA	
	HR (95% CI for HR)	*P* value	HR (95% CI for HR)	*P* value
ATP4A	13 (1.1-140)	3.92*E*-02	1.2 (1-1.4)	4.51*E*-02
CCDC73	29 (2.4-360)	8.45*E*-03	2.1 (1.3-3.5)	3.69*E*-03
CNTNAP4	35 (1.5-850)	2.75*E*-02	1.1 (1-1.3)	4.09*E*-02
CRB1	0.014 (0.00019-0.98)	4.88*E*-02	0.83 (0.68-1)	4.85*E*-02
EWSR1	5.4 (1.7-18)	5.05*E*-03	10 (1.9-54)	6.87*E*-03
FCGR3B	0.2 (0.043-0.9)	3.59*E*-02	0.77 (0.62-0.95)	1.37*E*-02
FRYL	0.053 (0.005-0.56)	1.48*E*-02	0.24 (0.083-0.7)	9.17*E*-03
GDF7	42 (1.6-1100)	2.44*E*-02	1.4 (1.1-1.9)	2.3*E*-02
GSG1L	520 (19-14000)	2.14*E*-04	1.2 (1-1.4)	3.33*E*-02
PRDM14	20 (1.4-270)	2.58*E*-02	1.2 (1-1.4)	2.3*E*-02
PRKDC	0.59 (0.4-0.86)	6.62*E*-03	0.41 (0.21-0.8)	9.25*E*-03
RER1	3.3 (1.2-8.9)	1.91*E*-02	6 (1.5-24)	1.12*E*-02
SYNPO	0.02 (0.00046-0.86)	4.15*E*-02	0.46 (0.26-0.81)	7.35*E*-03
XKR8	21 (1.4-300)	2.71*E*-02	3.7 (1.2-12)	2.55*E*-02
ZNF284	54 (1.4-2200)	3.37*E*-02	2.5 (1-6.1)	4.38*E*-02
ZNF680	2.3 (1.1-4.7)	2.48*E*-02	2.1 (1-4)	3.65*E*-02
ZNF780B	27 (1.8-390)	1.63*E*-02	2.7 (1.3-5.6)	9.42*E*-03

**Table 3:** Univariate and multivariate Cox regression analyses of the signature genes and overall survival.

Characters	Ch.description	Univariate analysis		Multivariate analysis	
		HR (95% CI for HR)	*P* value	HR (95% CI for HR)	*P* value
GEO					
Sex	Female/male	1.43 (0.76-2.7)	2.58*E*-01	1.13 (0.82-1.55)	3.92*E*-02
Age	>70/<=70	1.15 (1.02-1.14)	9.03*E*-03	1.17 (1.06-1.59)	2.91*E*-02
Invasiveness	Yes/no	2.32 (1.46-3.92)	1.64*E*-03	1.35 (0.65-2.83)	4.23*E*-02
Intravesical therapy	Yes/no	0.67 (0.48-0.93)	1.59*E*-02	0.91 (0.66-1.25)	4.57*E*-01
Stage	0-III	1.79 (1.37-2.23)	2.23*E*-04	1.39 (1.05-1.86)	2.38*E*-03
Grade	Low/high	2.76 (1.68-4.57)	1.97*E*-03	1.21 (0.91-1.62)	3.93*E*-02
Event	Yes/no	0.92 (0.51-1.6)	7.76*E*-01	0.46 (0.37-1.99)	1.79*E*-02
Specific survival	Alive/dead	13.02 (7.54-24)	6.62*E*-04	11.79 (10.94-17.38)	2.32*E*-03
Risk score	Low/high	4.45 (4.32-5.32)	3.15*E*-07	4.02 (4.99-8.04)	2.46*E*-04
TCGA					
Sex	Female/male	1.12 (0.73-1.68)	7.04*E*-03	2.95 (0.72-12.04)	1.355*E*-02
Age	>70/<=70	1.36 (0.76-2.35)	3.77*E*-02	1.22 (0.53-2.79)	6.42*E*-02
Height	>170/≤170 cm	1.24 (0.99-2.5)	2.43*E*-03	1.09 (1.03-1.16)	4.43*E*-03
Weight	>80/≤80	0.99 (0.98-1.07)	1.49*E*-02	0.97 (0.93-1.05)	2.97*E*-02
Diagnosis age	Yes/no	0.62 (0.33-1.89)	4.16*E*-02	0.83 (0.36-1.90)	6.64*E*-02
Stage	II/III	1.62 (1.3-2.11)	2.31*E*-05	1.5 (0.92-2.58)	9.92*E*-04
Grade	High/low	4.75 (3.63-7.91)	9.92*E*-02	3.8 (2.88-8.34)	1.87*E*-01
Noninvasive history	Yes/no	0.81 (0.63-1.87)	1.04*E*-02	0.83 (0.43-1.57)	5.68*E*-02
Smoking	No/<15 yr/>15 yr	1.41 (1.12-1.85)	7.47*E*-03	1.20 (0.67-2.15)	5.53*E*-03
Risk score	High/low	3.93 (3.34-6.35)	3.57*E*-06	3.11 (3.09-4.24)	8.42*E*-04

**Table 4:** The pathway enriched for the 17-gene signature based on four pathway databases.

Index	Name of pathway	*P* value
1	BIOCARTA_FAS_PATHWAY	9.42*E*-05
2	BIOCARTA_G_PATHWAY	2.23*E*-04
3	BIOCARTA_G2_PATHWAY	5.99*E*-05
4	BIOCARTA_HIVNEF_PATHWAY	3.55*E*-04
5	BIOCARTA_TNFR_PATHWAY	2.04*E*-04
6	BIOCARTA_TNFR1_PATHWAY	8.8*E*-05
7	KEGG_CELL_CYCLE	1.61*E*-03
8	KEGG_LEISHMANIA_INFECTION	5.17*E*-04
9	KEGG_NATURAL_KILLER_CELL_MEDIATED_CYTOTOXICITY	1.79*E*-03
10	KEGG_NON_HOMOLOGOUS_END_JOINING	1.7*E*-05
11	KEGG_OXIDATIVE_PHOSPHORYLATION	1.77*E*-03
12	KEGG_SYSTEMIC_LUPUS_ERYTHEMATOSUS	1.9*E*-03
13	KEGG_TGF_BETA_SIGNALING_PATHWAY	7.61*E*-04
14	PID_AR_PATHWAY	3.93*E*-04
15	PID_BARD_PATHWAY	3.36*E*-07
16	PID_BARD1PATHWAY	3.36*E*-07
17	PID_DNA_PK_PATHWAY	2.61*E*-05
18	PID_DNAPK_PATHWAY	2.61*E*-05
19	PID_PI3KCIAKTPATHWAY	1.29*E*-04
20	PID_PIKCI_AKT_PATHWAY	1.29*E*-04
21	REACTOME_DNA_REPAIR	1.16*E*-03
22	REACTOME_DOUBLE_STRAND_BREAK_REPAIR	5.02*E*-05
23	REACTOME_ION_CHANNEL_TRANSPORT	3.08*E*-04
24	REACTOME_ION_TRANSPORT_BY_P_TYPE_ATPASES	1.14*E*-04

## Data Availability

The raw data of RNA-seq and clinical data from TCGA and GEO datasets are available in the Supplemental Files.

## Supplementary Materials

Supplementary Materials Supplementary Table S1 gives the GO BP terms for the 17-gene signature with the *P* value less than 0.002.
