# Supplementary material for: Identification of an Individualized Prognostic Signature Based on the RWSR Model in Early-Stage Bladder Carcinoma
Source: Biomed Res Int. 2020 Jun 4;2020:9186546. doi: 10.1155/2020/9186546 (PMC7293744; doi:10.1155/2020/9186546)
Supplement: Supplementary Materials — Supplementary Table S1 gives the GO BP terms for the 17-gene signature with the P value less than 0.002. [file 9186546.f1.docx]

**Supplementary**

Table S1 The GO BP term for 17-gene signature (P-value < 0.002)

|  | **Term** | **Pvalue.FDR** |
| --- | --- | --- |
| **1** | GO_CELL_FATE_COMMITMENT | 3.65E-06 |
| **2** | GO_NEGATIVE_REGULATION_OF_FIBROBLAST_GROWTH_FACTOR_RECEPTOR_SIGNALING_PATHWAY | 1.2E-05 |
| **3** | GO_NEGATIVE_REGULATION_OF_CELLULAR_SENESCENCE | 1.2E-05 |
| **4** | GO_LYMPHOID_PROGENITOR_CELL_DIFFERENTIATION | 1.2E-05 |
| **5** | GO_SODIUM_ION_EXPORT | 1.44E-05 |
| **6** | GO_PHOSPHOLIPID_SCRAMBLING | 1.44E-05 |
| **7** | GO_CELLULAR_POTASSIUM_ION_HOMEOSTASIS | 1.44E-05 |
| **8** | GO_ESTABLISHMENT_OR_MAINTENANCE_OF_TRANSMEMBRANE_ELECTROCHEMICAL_GRADIENT | 1.7E-05 |
| **9** | GO_PATHWAY_RESTRICTED_SMAD_PROTEIN_PHOSPHORYLATION | 1.7E-05 |
| **10** | GO_PEPTIDYL_ARGININE_METHYLATION | 1.7E-05 |
| **11** | GO_FOREBRAIN_MORPHOGENESIS | 1.98E-05 |
| **12** | GO_SPINAL_CORD_ASSOCIATION_NEURON_DIFFERENTIATION | 1.98E-05 |
| **13** | GO_MORPHOGENESIS_OF_AN_EPITHELIAL_FOLD | 2.28E-05 |
| **14** | GO_T_CELL_LINEAGE_COMMITMENT | 2.28E-05 |
| **15** | GO_V_D_J_RECOMBINATION | 2.28E-05 |
| **16** | GO_NEGATIVE_REGULATION_OF_CELL_AGING | 2.96E-05 |
| **17** | GO_REGULATION_OF_DNA_METHYLATION | 2.96E-05 |
| **18** | GO_REGULATION_OF_SYNAPTIC_TRANSMISSION_DOPAMINERGIC | 2.96E-05 |
| **19** | GO_CELL_DEVELOPMENT | 3.1E-05 |
| **20** | GO_POTASSIUM_ION_HOMEOSTASIS | 3.33E-05 |
| **21** | GO_PEPTIDYL_ARGININE_MODIFICATION | 3.33E-05 |
| **22** | GO_CELLULAR_SODIUM_ION_HOMEOSTASIS | 3.72E-05 |
| **23** | GO_DORSAL_SPINAL_CORD_DEVELOPMENT | 4.13E-05 |
| **24** | GO_SOMATIC_RECOMBINATION_OF_IMMUNOGLOBULIN_GENE_SEGMENTS | 4.56E-05 |
| **25** | GO_ACTIVIN_RECEPTOR_SIGNALING_PATHWAY | 5.02E-05 |
| **26** | GO_REGULATION_OF_FIBROBLAST_GROWTH_FACTOR_RECEPTOR_SIGNALING_PATHWAY | 6.51E-05 |
| **27** | GO_POSTSYNAPTIC_MEMBRANE_ORGANIZATION | 6.51E-05 |
| **28** | GO_DEVELOPMENTAL_PROGRAMMED_CELL_DEATH | 7.05E-05 |
| **29** | GO_REGULATION_OF_CELLULAR_SENESCENCE | 7.05E-05 |
| **30** | GO_APOPTOTIC_CELL_CLEARANCE | 7.61E-05 |
| **31** | GO_SOMATIC_DIVERSIFICATION_OF_IMMUNOGLOBULINS | 8.19E-05 |
| **32** | GO_TELOMERE_CAPPING | 8.8E-05 |
| **33** | GO_NEGATIVE_REGULATION_OF_PRODUCTION_OF_MOLECULAR_MEDIATOR_OF_IMMUNE_RESPONSE | 8.8E-05 |
| **34** | GO_BLASTOCYST_FORMATION | 8.8E-05 |
| **35** | GO_POTASSIUM_ION_IMPORT | 8.8E-05 |
| **36** | GO_REGULATION_OF_SYNAPTIC_TRANSMISSION_GABAERGIC | 8.8E-05 |
| **37** | GO_SODIUM_ION_HOMEOSTASIS | 9.42E-05 |
| **38** | GO_HOMEOSTASIS_OF_NUMBER_OF_CELLS_WITHIN_A_TISSUE | 9.42E-05 |
| **39** | GO_EYE_PHOTORECEPTOR_CELL_DEVELOPMENT | 1.01E-04 |
| **40** | GO_SOMATIC_CELL_DNA_RECOMBINATION | 1.07E-04 |
| **41** | GO_EMBRYO_DEVELOPMENT_ENDING_IN_BIRTH_OR_EGG_HATCHING | 1.14E-04 |
| **42** | GO_REGULATION_OF_CELL_AGING | 1.14E-04 |
| **43** | GO_PLASMA_MEMBRANE_ORGANIZATION | 1.15E-04 |
| **44** | GO_PROTEIN_DESTABILIZATION | 1.21E-04 |
| **45** | GO_BRAIN_MORPHOGENESIS | 1.21E-04 |
| **46** | GO_NEUROMUSCULAR_JUNCTION_DEVELOPMENT | 1.29E-04 |
| **47** | GO_POSITIVE_REGULATION_OF_PROTEIN_LOCALIZATION_TO_CELL_PERIPHERY | 1.29E-04 |
| **48** | GO_REGULATION_OF_MEMBRANE_LIPID_DISTRIBUTION | 1.36E-04 |
| **49** | GO_ATP_HYDROLYSIS_COUPLED_TRANSMEMBRANE_TRANSPORT | 1.36E-04 |
| **50** | GO_PHAGOCYTOSIS_ENGULFMENT | 1.52E-04 |
| **51** | GO_SPLEEN_DEVELOPMENT | 1.52E-04 |
| **52** | GO_EMBRYO_IMPLANTATION | 1.52E-04 |
| **53** | GO_DEVELOPMENTAL_PROCESS_INVOLVED_IN_REPRODUCTION | 1.56E-04 |
| **54** | GO_PHOTORECEPTOR_CELL_DEVELOPMENT | 1.6E-04 |
| **55** | GO_SOMATIC_DIVERSIFICATION_OF_IMMUNE_RECEPTORS | 1.68E-04 |
| **56** | GO_RECEPTOR_CLUSTERING | 1.77E-04 |
| **57** | GO_EYE_PHOTORECEPTOR_CELL_DIFFERENTIATION | 1.86E-04 |
| **58** | GO_CELLULAR_PROCESS_INVOLVED_IN_REPRODUCTION_IN_MULTICELLULAR_ORGANISM | 2.02E-04 |
| **59** | GO_THYMOCYTE_AGGREGATION | 2.14E-04 |
| **60** | GO_IMMUNOGLOBULIN_PRODUCTION | 2.14E-04 |
| **61** | GO_THYMUS_DEVELOPMENT | 2.23E-04 |
| **62** | GO_REGULATION_OF_ESTABLISHMENT_OF_PROTEIN_LOCALIZATION_TO_PLASMA_MEMBRANE | 2.23E-04 |
| **63** | GO_REGULATION_OF_IMMUNOGLOBULIN_PRODUCTION | 2.33E-04 |
| **64** | GO_POSITIVE_REGULATION_OF_ACTIN_FILAMENT_BUNDLE_ASSEMBLY | 2.33E-04 |
| **65** | GO_MEMBRANE_INVAGINATION | 2.43E-04 |
| **66** | GO_POSITIVE_REGULATION_OF_PATHWAY_RESTRICTED_SMAD_PROTEIN_PHOSPHORYLATION | 2.43E-04 |
| **67** | GO_NEURON_DEVELOPMENT | 2.46E-04 |
| **68** | GO_RESPONSE_TO_GAMMA_RADIATION | 2.53E-04 |
| **69** | GO_PHOTORECEPTOR_CELL_DIFFERENTIATION | 2.64E-04 |
| **70** | GO_POSITIVE_REGULATION_OF_FIBROBLAST_PROLIFERATION | 2.85E-04 |
| **71** | GO_ANATOMICAL_STRUCTURE_HOMEOSTASIS | 2.97E-04 |
| **72** | GO_CELL_DIFFERENTIATION_IN_SPINAL_CORD | 2.97E-04 |
| **73** | GO_EXECUTION_PHASE_OF_APOPTOSIS | 2.97E-04 |
| **74** | GO_ACTIN_CYTOSKELETON_REORGANIZATION | 3.08E-04 |
| **75** | GO_SMAD_PROTEIN_SIGNAL_TRANSDUCTION | 3.31E-04 |
| **76** | GO_REGULATION_OF_PATHWAY_RESTRICTED_SMAD_PROTEIN_PHOSPHORYLATION | 3.68E-04 |
| **77** | GO_BLASTOCYST_DEVELOPMENT | 3.8E-04 |
| **78** | GO_SOMITOGENESIS | 4.06E-04 |
| **79** | GO_PRODUCTION_OF_MOLECULAR_MEDIATOR_OF_IMMUNE_RESPONSE | 4.06E-04 |
| **80** | GO_REGULATION_OF_BEHAVIOR | 4.33E-04 |
| **81** | GO_SOMATIC_STEM_CELL_POPULATION_MAINTENANCE | 4.6E-04 |
| **82** | GO_RESPONSE_TO_ACTIVITY | 4.74E-04 |
| **83** | GO_NON_RECOMBINATIONAL_REPAIR | 5.03E-04 |
| **84** | GO_POSITIVE_REGULATION_OF_TYPE_I_INTERFERON_PRODUCTION | 5.17E-04 |
| **85** | GO_INTRINSIC_APOPTOTIC_SIGNALING_PATHWAY_IN_RESPONSE_TO_DNA_DAMAGE | 5.17E-04 |
| **86** | GO_CELL_FATE_SPECIFICATION | 5.17E-04 |
| **87** | GO_REGULATION_OF_PLASMA_MEMBRANE_ORGANIZATION | 5.32E-04 |
| **88** | GO_HISTONE_METHYLATION | 5.94E-04 |
| **89** | GO_REGULATION_OF_ACTIN_FILAMENT_BUNDLE_ASSEMBLY | 5.94E-04 |
| **90** | GO_RETROGRADE_VESICLE_MEDIATED_TRANSPORT_GOLGI_TO_ER | 6.09E-04 |
| **91** | GO_NEURON_DIFFERENTIATION | 6.24E-04 |
| **92** | GO_SOMITE_DEVELOPMENT | 6.25E-04 |
| **93** | GO_CELLULAR_COMPONENT_MORPHOGENESIS | 6.61E-04 |
| **94** | GO_MEMBRANE_ORGANIZATION | 6.72E-04 |
| **95** | GO_REGULATION_OF_FIBROBLAST_PROLIFERATION | 6.75E-04 |
| **96** | GO_EMBRYO_DEVELOPMENT | 6.96E-04 |
| **97** | GO_SODIUM_ION_TRANSMEMBRANE_TRANSPORT | 7.44E-04 |
| **98** | GO_B_CELL_DIFFERENTIATION | 7.97E-04 |
| **99** | GO_GLAND_DEVELOPMENT | 8.17E-04 |
| **100** | GO_MIDBRAIN_DEVELOPMENT | 8.34E-04 |
| **101** | GO_SEGMENTATION | 8.34E-04 |
| **102** | GO_ANATOMICAL_STRUCTURE_FORMATION_INVOLVED_IN_MORPHOGENESIS | 8.53E-04 |
| **103** | GO_SIGNAL_TRANSDUCTION_IN_RESPONSE_TO_DNA_DAMAGE | 8.91E-04 |
| **104** | GO_RESPONSE_TO_BMP | 9.1E-04 |
| **105** | GO_NEGATIVE_REGULATION_OF_CELL_CYCLE_G_S_PHASE_TRANSITION | 9.1E-04 |
| **106** | GO_CELLULAR_MONOVALENT_INORGANIC_CATION_HOMEOSTASIS | 9.3E-04 |
| **107** | GO_NEURAL_TUBE_FORMATION | 9.3E-04 |
| **108** | GO_MITOTIC_DNA_INTEGRITY_CHECKPOINT | 9.5E-04 |
| **109** | GO_HEMATOPOIETIC_PROGENITOR_CELL_DIFFERENTIATION | 9.7E-04 |
| **110** | GO_REGULATION_OF_IMMUNE_EFFECTOR_PROCESS | 9.71E-04 |
| **111** | GO_GLAND_MORPHOGENESIS | 9.9E-04 |
| **112** | GO_REGULATION_OF_SMOOTH_MUSCLE_CELL_PROLIFERATION | 1.03E-03 |
| **113** | GO_REGULATION_OF_CIRCADIAN_RHYTHM | 1.05E-03 |
| **114** | GO_G_DNA_DAMAGE_CHECKPOINT | 1.05E-03 |
| **115** | GO_POSITIVE_REGULATION_OF_TRANSMEMBRANE_RECEPTOR_PROTEIN_SERINE_THREONINE_KINASE_SIGNALING_PATHWAY | 1.05E-03 |
| **116** | GO_NEGATIVE_REGULATION_OF_IMMUNE_EFFECTOR_PROCESS | 1.05E-03 |
| **117** | GO_PROTEIN_ALKYLATION | 1.07E-03 |
| **118** | GO_REGULATION_OF_PRODUCTION_OF_MOLECULAR_MEDIATOR_OF_IMMUNE_RESPONSE | 1.07E-03 |
| **119** | GO_XENOPHAGY | 1.09E-03 |
| **120** | GO_HYDROGEN_ION_TRANSMEMBRANE_TRANSPORT | 1.11E-03 |
| **121** | GO_SPINAL_CORD_DEVELOPMENT | 1.14E-03 |
| **122** | GO_TELOMERE_ORGANIZATION | 1.14E-03 |
| **123** | GO_REGULATION_OF_TYPE_I_INTERFERON_PRODUCTION | 1.25E-03 |
| **124** | GO_ENDOMEMBRANE_SYSTEM_ORGANIZATION | 1.25E-03 |
| **125** | GO_NEGATIVE_REGULATION_OF_CELLULAR_RESPONSE_TO_GROWTH_FACTOR_STIMULUS | 1.48E-03 |
| **126** | GO_LOCALIZATION_WITHIN_MEMBRANE | 1.51E-03 |
| **127** | GO_MONOVALENT_INORGANIC_CATION_HOMEOSTASIS | 1.53E-03 |
| **128** | GO_T_CELL_DIFFERENTIATION | 1.58E-03 |
| **129** | GO_B_CELL_ACTIVATION | 1.74E-03 |
| **130** | GO_TUBE_FORMATION | 1.74E-03 |
| **131** | GO_MACROMITOPHAGY | 1.79E-03 |
| **132** | GO_BRANCHING_MORPHOGENESIS_OF_AN_EPITHELIAL_TUBE | 1.79E-03 |
| **133** | GO_REGULATION_OF_DEFENSE_RESPONSE_TO_VIRUS_BY_HOST | 1.79E-03 |
| **134** | GO_MAINTENANCE_OF_CELL_NUMBER | 1.82E-03 |
| **135** | GO_MITOTIC_CELL_CYCLE_CHECKPOINT | 1.82E-03 |
| **136** | GO_MORPHOGENESIS_OF_EMBRYONIC_EPITHELIUM | 1.85E-03 |
| **137** | GO_EYE_MORPHOGENESIS | 1.85E-03 |
| **138** | GO_HYDROGEN_TRANSPORT | 1.87E-03 |
| **139** | GO_ESTABLISHMENT_OR_MAINTENANCE_OF_CELL_POLARITY | 1.9E-03 |
| **140** | GO_SODIUM_ION_TRANSPORT | 1.9E-03 |
| **141** | GO_NEURON_PROJECTION_DEVELOPMENT | 1.93E-03 |
| **142** | GO_RESPONSE_TO_IONIZING_RADIATION | 1.99E-03 |
